# Deep Sequencing of Pyrethroid-Resistant Bed Bugs Reveals Multiple Mechanisms of Resistance within a Single Population

**DOI:** 10.1371/journal.pone.0026228

**Published:** 2011-10-19

**Authors:** Zach N. Adelman, Kathleen A. Kilcullen, Reina Koganemaru, Michelle A. E. Anderson, Troy D. Anderson, Dini M. Miller

**Affiliations:** 1 Fralin Life Science Institute and Department of Entomology, Virginia Tech, Blacksburg, Virginia, United States of America; 2 Dodson Urban Pest Management Laboratory and Department of Entomology, Virginia Tech, Blacksburg, Virginia, United States of America; New Mexico State University, United States of America

## Abstract

A frightening resurgence of bed bug infestations has occurred over the last 10 years in the U.S. and current chemical methods have been inadequate for controlling this pest due to widespread insecticide resistance. Little is known about the mechanisms of resistance present in U.S. bed bug populations, making it extremely difficult to develop intelligent strategies for their control. We have identified bed bugs collected in Richmond, VA which exhibit both *kdr*-type (L925I) and metabolic resistance to pyrethroid insecticides. Using LD_50_ bioassays, we determined that resistance ratios for Richmond strain bed bugs were ∼5200-fold to the insecticide deltamethrin. To identify metabolic genes potentially involved in the detoxification of pyrethroids, we performed deep-sequencing of the adult bed bug transcriptome, obtaining more than 2.5 million reads on the 454 titanium platform. Following assembly, analysis of newly identified gene transcripts in both Harlan (susceptible) and Richmond (resistant) bed bugs revealed several candidate cytochrome P450 and carboxylesterase genes which were significantly over-expressed in the resistant strain, consistent with the idea of increased metabolic resistance. These data will accelerate efforts to understand the biochemical basis for insecticide resistance in bed bugs, and provide molecular markers to assist in the surveillance of metabolic resistance.

## Introduction

The common bed bug, *Cimex lectularius* (Hemiptera: Cimicidae) was a well established pest in the United States, and other developed nations, at the turn of the 20^th^ century. However, due to the widespread use of DDT, bed bugs were essentially eradicated from U.S. homes and apartments by the 1950s. While DDT was initially effective for bed bug control, resistance to the cyclodienes was well documented among different bed bug populations by 1958 [1], [2]. The National Pest Control Association's initial recommendation for combating DDT-resistant bed bug populations was malathion (as reviewed by [3]). Yet, while malathion further reduced the incidence of bed bug infestations in developed nations, bed bugs had developed resistance to organophosphate insecticides, including malathion by the 1960s [4].

In recent years, the incidence of bed bug infestations has increased rapidly within the U.S. As of 2010, bed bug infestations have been reported in all 50 states. While there have been many hypotheses regarding the cause of the bed bug resurgence, the cause is at least partially explained by bed bug resistance to insecticides, in this case, those in the pyrethroid class [5], [6], [7], [8]. Attempts to characterize the mechanisms of resistance in these resurgent bed bug populations have followed. Yoon et al. [7] found that resistance to deltamethrin in a bed bug strain from New York City was the result of two point mutations (V419L and L925I) in the alpha-subunit of the voltage-gated sodium channel, which is the target of pyrethroid insecticides. Subsequently, Zhu et al. [8] screened 110 bed bug strains collected from different regions of the U.S. and found that 88% of those populations had either one or both of these target site mutations, indicating that this target site resistance is widespread in reemerging bed bug populations.

While a target site mutation, or *kdr* resistance, was identified as the primary mechanism of resistance in the previously noted studies, other mechanisms of resistance such as enhanced detoxification enzyme activity have not been as well documented in modern bed bug strains. The New York strain analyzed by Yoon et al [7] showed no elevated activity in any of the major insecticide-metabolizing enzyme systems; glutathione-*S*-transferases (GST), carboxylesterases (CE), and cytochrome P450 monooxygenases (P450) using model substrates. However, Romero et al. [9] observed a decrease in resistance to pyrethroid insecticides using piperonyl butoxide (PBO), an inhibitor of P450 activity, in bed bugs collected in Cincinnati, OH and Worcester, MA. This suggests that detoxification is an important mechanism of resistance in these strains, though other resistance mechanisms must also be present. Likewise, Bai et al [10] found increased transcript levels for a cytochrome P450 (CYP9) in bed bugs collected in Columbus, OH, compared with those of susceptible (non-exposed) strains.

Biochemical analyses of resistance phenotypes are labor-intensive and costly. As such, PCR-based methods are preferred to screen large numbers of individuals/populations for a defined trait such as a nucleotide polymorphism or for differences in gene expression levels, which are strongly associated with a resistance phenotype. While such a diagnostic PCR method has been developed for surveying target site resistance [8], no such assays have been associated with metabolic resistance in bed bugs. In this paper, we present both biochemical and genetic evidence that bed bug populations collected in Richmond, VA carry both target site and metabolic resistance traits, and these correlate with ∼5200-fold resistance to the pyrethroid deltamethrin. Further, we have identified through deep sequencing a large cohort of full-length P450, GST and CE-encoding sequences, several of which are significantly upregulated in resistant bed bugs. These sequences will aid in surveillance efforts to detect and monitor metabolic resistance in resurgent bed bug populations and enable studies involving the biochemical characterization of precisely how bed bugs metabolize and inactivate insecticides.

## Materials and Methods

### Bed bug collection and maintenance

Pyrethroid-resistant field-strain bed bugs (several hundred adult males, females, nymphs, and eggs) were collected from a group home in Richmond, VA, in the fall of 2008 and transported to the Dodson Urban Pest Management Laboratory in Blacksburg, VA. A pyrethroid-susceptible strain was acquired from Dr. Harold Harlan of the National Pest Management Association, Fairfax, VA, in February 2005. This strain had been originally collected from Ft. Dix, New Jersey and maintained in colony (feeding on Dr. Harlan) since 1973. These strains were designated as “Richmond” and “Harlan”, respectively. All bed bugs were kept in plastic rearing containers with cardboard harborages in an environmental chamber with 27°C (26.1–27.3°C), 70% relative humidity, and a 12∶12 light and dark photoperiod. Blood was provided once a week either by a human volunteer during initial establishment of the Richmond strain [written informed consent was received for all participants; this study was approved by the Virginia Tech Institutional Review Board (Approval #06–165)] or using an artificial feeder containing chicken blood formulated with sodium citrate as an anti-coagulant (all experiments).

### Determination of resistance levels to pyrethroid insecticides

Injection bioassays were conducted to determine the susceptibility of each of the bed bug strains to pyrethroids. To determine LD_50_ values for bed bugs exposed to pyrethroids, technical grade pyrethroid insecticides (deltamethrin and β-cyfluthrin) (Sigma Aldrich, St. Louis, MO) were dissolved in DMSO. The DMSO was diluted to 10% with distilled water to reduce toxicity. Stock solutions of the highest concentrations were made at 500 µl each, and serially diluted 10-fold. Glass capillary needles were prepared by pulling Kwik-Fil Silicate Glass Capillaries with a P-2000 Laser micropipette puller. Each dilution (0.5 µl) of insecticide solution was injected into individual bed bugs from the left side of the ventral thorax between the second and third coxa. The lethal dosages (LD_50_ values) for each were calculated by counting mortality after 24 hours of application, and the data was analyzed using Polo Plus Probit and Logit Analysis computer software version 1.0.

### Detoxification Enzyme Activity Assays

General esterase activity was determined in adult bed bugs according to the method described by Zhu and Gao [11]. Each sample was homogenized in ice-cold 0.1 M phosphate buffer (pH 7.0) containing 0.3% (v/v) Triton X-100 (Sigma Aldrich, St. Louis, MO). After the homogenates were centrifuged at 10,000× g for 10 min. at 4°C, the supernatants were used as the enzyme source for measuring general esterase activities with α-naphthyl acetate (α-NA) and β-naphthyl acetate (β-NA) (Sigma Aldrich) as substrates. The absorbance was read using a SpectraMax M2 multimode microplate reader (Molecular Devices, Inc., Sunnyvale, CA) at 600 and 560 nm for α-NA and β-NA, respectively. Glutathione *S*-transferase activity was determined according to Zhu et al. [12] using 1-chloro-2, 4-dinitrobenzene (CDNB; Sigma Aldrich) as a substrate. The conjugation of glutathione towards CDNB was determined by recording the change in absorbance at 340 nm for CDNB for 1 min at 10-sec intervals using a multimode microplate reader. Non-enzymatic controls were performed in parallel to correct for non-enzymatic conjugation. Cytochrome P450-dependent *O*-deethylation activity was determined according to the method of Anderson and Zhu [13] using 7-ethoxycoumarin (7-EC) (Sigma Aldrich) as a substrate. The relative fluorescence units were measured using a multimode microplate reader at 465 nm while exciting at 390 nm. Total protein in each sample preparation was determined using the bicinchoninic acid assay with bovine serum albumin (Sigma Aldrich) as a standard. The measurement was performed on a multimode microplate reader at 560 nm.

### Extraction of mRNAs and preparation of cDNAs

Adult bed bugs were frozen in liquid nitrogen at seven days after the last molt and stored at −80°C until use. Bed bugs were homogenized and total RNA extracted using TRIzol (Invitrogen, Carlsbad, CA). Bed bug mRNA was enriched from the total RNA sample using the PolyATract mRNA Isolation System (Promega BioSciences, San Luis Obispo, CA). For 454 libraries, cDNA was synthesized using the Superscript Double-Stranded cDNA Synthesis kit (Invitrogen). For real-time qPCR, cDNA was synthesized using the High Capacity cDNA Reverse Transcription Kit (Applied Biosystems, Foster City, CA). Following elimination of the input RNA and purification, cDNA concentrations were determined using a NanoDrop ND-1000 Spectrophotometer (NanoDrop Technologies, Wilmington, DE) prior to use.

### High-throughput sequencing and bioinformatic analysis

Library preparation and sequencing were performed by the Virginia Bioinformatics Institute core facility using the Roche 454 (Titanium) platform. Raw sequencing reads from the Harlan (both male and female) and Richmond (male) strain bed bugs were assembled in the absence of a reference genome using the NEWBLER assembler either individually or together. Assembled contigs were converted into a BLAST database [14] and queried with P450, carboxylesterase (CE) and GST protein sequences from a selection of the currently sequenced insect genomes, including *A. gambiae*, *Ae. aegypti*, *D. melanogaster*, *P. humanus*, and *A. pisum*. Matching contigs were queried back against the raw reads and extended manually where possible. Manually curated *C. lectularius* contigs were used as queries to find related sequences in the assembled and unassembled datasets, as well as to confirm orthology with the respective protein family. The presence of putative P450, GST or CE domains was predicted using Pfam [15]. All alignments were performed using ClustalX in MEGA 4.0 [16]. Raw sequence files, the complete assembled contig set, as well as all manually assembled sequences are available at the Gene Expression Omnibus (Accession# GSE31025).

### Real-time PCR quantization of gene expression

For all genes, primer pairs were designed using Lasergene Primer Select (DNASTAR, Madison, WI), with the following criteria: length of 20–24 bps, melting temperature between 50–60°C, total product size around 150–250 bps, and a GC content close to 50%. (see Table S1 for a list of primers used in this study). qPCR reactions were performed using POWER SYBR Green PCR Master Mix (Applied Biosystems, Foster City, CA) on a StepOne Real-Time PCR system using the following conditions: 95°C (10 min) followed by 40 cycles of 95°C (15 sec), 51°C (30 sec), and 72°C (30 sec). Two reference genes (*C. lectularius* myosin heavy chain and alpha tubulin) were included with every run to normalize expression and control for plate effects. To verify the specificity of each qPCR reaction, all amplified products were confirmed through melt curve analysis and gel electrophoresis. Comparative cycle threshold (C_t_) analysis was performed to determine the relative expression of Richmond strain bed bugs compared with Harlan using Real-time StatMiner (Integromics®, Granada, Spain) with the moderated t-test (Limma) and Benjamani-Hochberg false discovery p-value adjustment.

## Results

### Bed bugs collected in Richmond, VA are highly resistant to pyrethroid insecticides

We previously demonstrated that bed bugs collected in Richmond, VA display a greatly extended time to mortality when exposed to formulated deltamethrin (0.06%) and permethrin (0.05%) [17]. To more precisely determine the level of resistance to pyrethroid insecticides, injection bioassays were conducted to determine the specific susceptibility of each bed bug strain. The lethal doses required to kill 50% (LD_50_) of bed bugs exposed to the pyrethroids deltamethrin or *β*-cyfluthrin are presented in Table 1. When mortality was calculated at 24 h following injection, the deltamethrin LD_50_ value for the Richmond strain bed bugs was almost 5200-fold greater than that of the laboratory strain. Similarly, the LD_50_ value calculated for the Richmond strain bed bugs exposed to *β*-cyfluthrin was 111-fold greater than that of the laboratory strain. Thus, we conclude that the Richmond strain bed bugs are highly resistant to these insecticides.

**Table 1:** Acute toxicities of deltamethrin and β-cyfluthrin to Richmond and Harlan strain bed bugs^a^.

Insecticide	Strain	N	LD_50_ (ng/insect)	95% CI	Slope±SE	RR^b^
Deltamethrin	Richmond	60	155	55.2–555.3	1.3±0.4	5,167^*^
	Harlan	90	0.03	8.8E-03–1.5E-01	1.2±0.3	
β-Cyfluthrin	Richmond	100	4.43	3.19–6.09	3.1±1.0	111^*^
	Harlan	90	0.04	2.5E-02–5.4E-02	3.2±0.7	

a Insecticide toxicities are presented as LD_50_ and their 95% confidence intervals (95% CI) in nanograms (ng) per insect, the dose at which 50% of the test populations were killed in a 24-h bioassay.

b Resistance ratios (RR) were calculated by RR = LD_50Richmond_/LD_50 Harlan_. The asterisk next to the ratios indicates a significant difference between the LD_50_ values for the Richmond and Harlan strains based on the non-overlapping 95% CI of the LD_50_ values.

### Identification of kdr in Richmond strain bed bugs

Yoon et al [7] sequenced the bed bug sodium channel gene and identified two amino acid substitutions in pyrethroid-resistant bed bugs collected in New York, V419L and L925I. To determine if these or any other substitutions were present in Richmond strain bed bugs, we sequenced the complete sodium channel coding region from both Richmond and Harlan strain bed bugs. Both Richmond and Harlan contained the wild-type V419, similar to the susceptible Florida strain described by Yoon et al [7]. However, Richmond strain bed bugs possessed the L925I substitution, while the susceptible Harlan strain did not. No other substitutions were found, with the exception of an A1925T conservative change found in the final cytoplasmic tail of both Harlan and Richmond strain genes.

### Characterization of detoxification enzyme activities from insecticide-susceptible and -resistant bed bug populations

**Figure 1:**
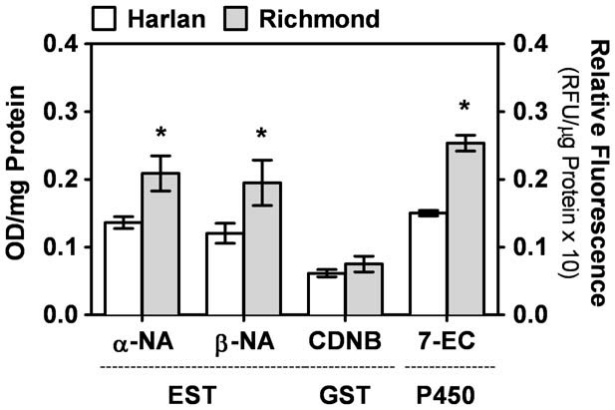
Comparison of general esterase (EST), glutathione *S*-transferase (GST), and cytochrome P450-dependent *O*-deethylation (P450) activities of insecticide-susceptible and-resistant bed bugs. Vertical bars indicate standard errors of the mean (*n* = 4), asterisks (*) denote that the means are significantly different between the two bed bug strains (two-tailed t-test, *P*<0.05).

To examine the possibility of metabolic resistance in Richmond strain bed bugs, we determined the general esterase, glutathione *S*-transferase, and cytochrome P450 monooxygenase activities of these detoxification enzymes in both the Harlan and Richmond strains (Fig. 1). We found that the general esterase activity of deltamethrin-resistant bed bugs was significantly increased (by 35% and 38% when α-NA and β-NA are used as substrates, respectively) compared to the insecticide-susceptible Harlan strain bed bugs (*P*<0.05). The cytochrome P450 *O*-deethylation activity of the Richmond strain bed bugs was also significantly enhanced by 41% compared to the insecticide-susceptible bed bugs (*P*<0.05). In contrast, glutathione *S*-transferase activity of deltamethrin-resistant bed bugs did not differ from that of the susceptible strain. These data suggest that increased esterase and P450 activities may play a role in detoxifying the pyrethroid deltamethrin.

### Identification of bed bug resistance genes through deep sequencing

**Figure 2:**
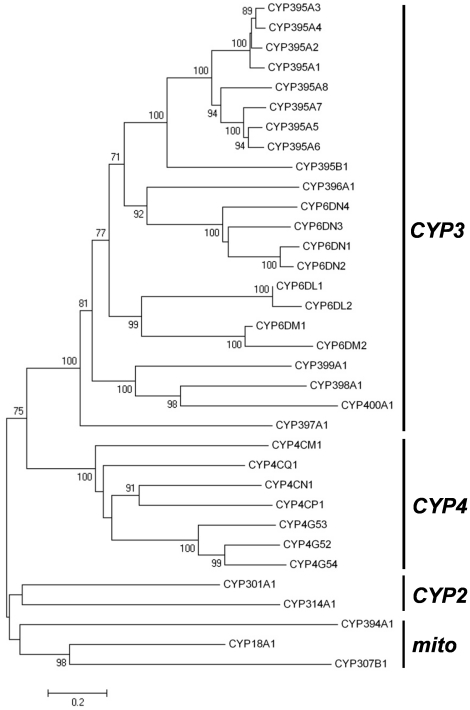
Phylogenetic relationship of bed bug P450s. Cytochrome P450 genes of the bed bug, *Cimex lectularius*. Predicted protein sequences were aligned using ClustalW; tree construction was performed using the Neighbor-joining method in MEGA 4.0. Bootstrap support is indicated where greater than 70%. Clades assigned to the CYP2, CYP3, CYP4 and mito clans are indicated. Family assignments and the naming of new families were performed by David Nelson as described [18].

At the time this project was initiated, little information was available for the sequences of bed bug genes which may play a role in insecticide detoxification. To obtain gene sequences for all abundantly expressed bed bug metabolic genes, we performed two 454 titanium runs using Harlan or Richmond strain cDNA, obtaining more than 2.5 million reads in total (Table 2). Harlan and Richmond strain reads were assembled together, yielding more than 14,000 contigs greater than 500 bp, with an N_50_ contig size greater than 1,200 bp (Table 2). From these contigs, we identified at least 34 members of the cytochrome P450 family. Most of these sequences were full length, as determined by the presence of start and termination codons spanning an ORF typical of P450 genes (avg ORF size was 487 a.a., median was 507). Only five sequences [*cyp18a1* (282 a.a.), *cyp400a1* (287 a.a.), *cyp301a1* (296 a.a.), *cyp6dm2* (358 a.a.), *cyp6dl2* (406 a.a.)] were relatively incomplete, with all other predicted protein sequences greater than 470 amino acids. The relative completeness of P450 coding regions allowed us to assign each P450 gene into a clade and family according to the Committee on Standardized Cytochrome P450 Nomenclature [18]. Of the 34 assembled P450 contigs, 22 derived from the CYP3 clan (Fig. 2). This included at least 8 new members of the CYP6 family, as well as new families CYP395 (at least 9 contigs), CYP 396 (1), CYP 397 (1), CYP 398 (1), CYP399 (1), and CYP 400 (1). We also identified members of clans CYP4 (7 contigs), CYP2 (2 contigs), and MITO (3 contigs) (Fig. 2).

**Table 2:** Deep sequencing of Richmond and Harlan strain bed bug transcriptomes using the Roche 454 titanium platform.

	Harlan strain (male and female adults)	Richmond strain (male adults)	Merged
Total # of reads	1,153,077	1,379,221	2,532,298
Total # of bases	464,907,362	500,792,578	965,699,940
# Aligned reads	1,073,218 (93.07%)	1,214,095 (88.03%)	2,247,916 (88.77%)
# Large contigs (>500 bp)	12,294	10,193	14,605
Largest contig	9,270	4,790	9,943
Avg. contig	1,110	950	1,120
N_50_ contig	1,231	1,002	1,276
Total contigs	22,940	23,114	32,819

**Figure 3:**
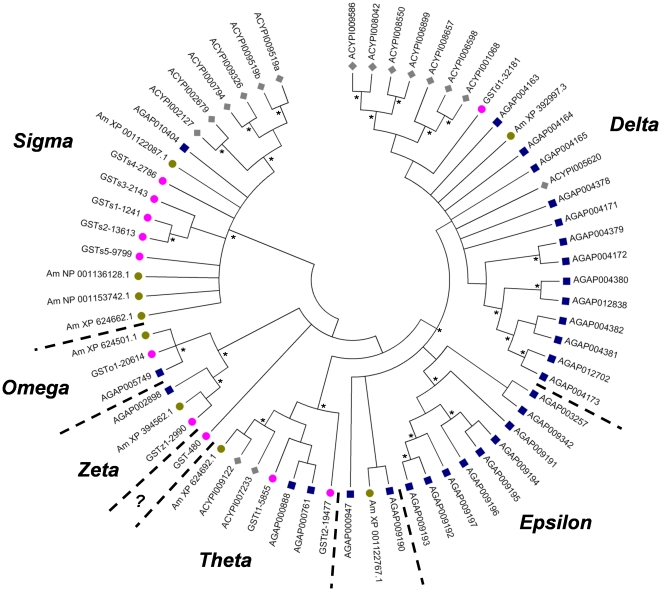
Phylogenetic comparison of bed bug GST genes with other insects. Glutathione *S*-transferase (GST) genes of the bed bug, *Cimex lectularius* (pink circle), compared with published sequences from the pea aphid *Acyrthosiphon pisum* (gray diamond), honey bee, *Apis mellifera* (green circle), and the malaria mosquito, *Anopheles gambiae* (blue square). Predicted protein sequences were aligned using ClustalW; tree construction was performed using the Neighbor-joining method in MEGA 4.0. All branches with less than 50% bootstrap support were collapsed, support greater than 80% is indicated (*). Classification is based on Ranson et al [27].

Ten contigs encoding full-length glutathione *S*-transferase ORFs were identified and aligned with those of other insect species (Fig. 3). Surprisingly, only a single member of the delta-epsilon class was identified. This contrasts with 22 members from the malaria mosquito and 8 from pea aphid. Similar to both the pea aphid and honey bee [19], [20], bed bugs have undergone a lineage-specific expansion of the sigma class GSTs, the significance of which remains unknown (Fig. 3). We also identified at least 24 contigs corresponding to bed bug carboxylesterase (CE) genes. Lower coverage of these genes combined with much higher variability from other insect sequences made assignment to specific clades more challenging. A preliminary phylogeny is shown in Fig. S1, but we stress that higher coverage sequencing, as well as additional sequences from more closely related hemipterans will be required to properly establish the relationship of these sequences with those of other insects. Of note are at least seven bed bug CE contigs whose ORF is predicted to encode a signal peptide [21], and thus may be important in the metabolism of pyrethroid insecticides.

### mRNA upregulation of metabolic genes in pyrethroid-resistant Richmond strain bed bugs

**Figure 4:**
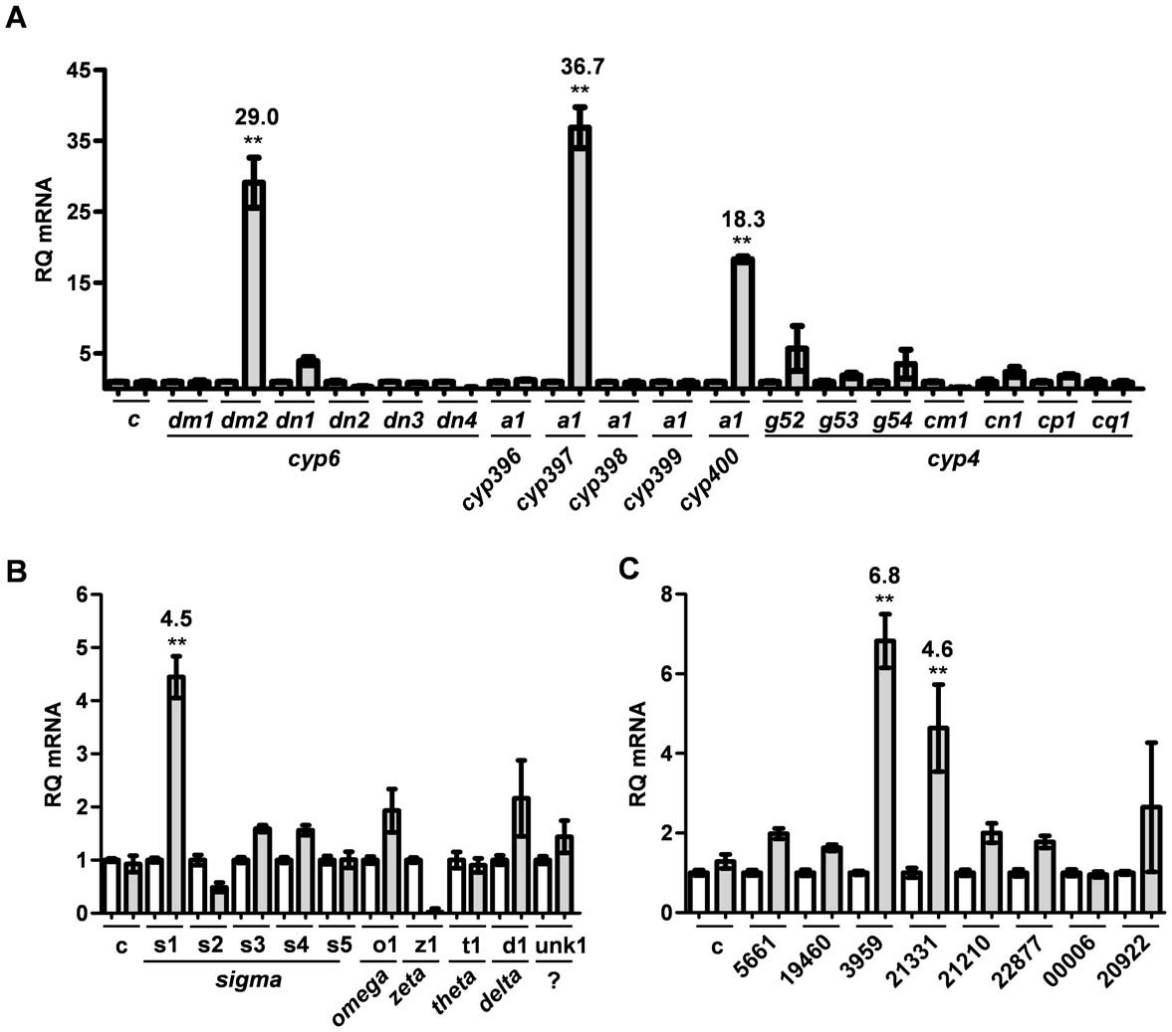
Relative expression of metabolic genes in Harlan and Richmond strain bed bugs. Real-time quantitative PCR was performed to measure the relative expression levels of (**A**) P450, (**B**) GST, and (**C**) CE transcripts in the pyrethroid-resistant Richmond strain as compared with the susceptible Harlan strain. Each bar represents the mean of two biological replicates, each performed in triplicate. Error bars indicate the standard deviation from the mean. All samples were normalized to the expression of alpha-tubulin. A second control gene [myosin heavy-chain, indicated by (c)] was included to verify plate-to-plate consistency. Harlan (white) and Richmond (shaded) are indicated for each gene, with Harlan set to 1 for all samples. Fold change is indicated above each bar where 2-fold or greater for all statistically significant samples (** = p<0.01).

In order to determine if any of the identified P450, GST or CE (signal peptide-containing only) genes were transcriptionally upregulated in pyrethroid-resistant bed bugs, we performed quantitative real-time PCR on cDNA generated from Harlan or Richmond strain adult male bed bugs. Analysis of the expression of P450 genes revealed that genes *cyp397a1* (>36 fold), *cyp6dm2* (>29 fold) and *cyp400a1* (>18-fold) were all significantly over-expressed in Richmond, pyrethroid-resistant bed bugs as compared to the Harlan control (Fig. 4A). Extremely high similarity between CYP395 family members, as well as between contigs *cyp6dl1* and *cyp6dl2* prevented analysis of their expression using SYBR® green-based qPCR assays. More specific assays employing hydrolysis probes would likely be needed in order to ascertain any expression changes in these genes. qPCR amplification of GST-encoding gene transcripts revealed that only *GSTs1* was significantly upregulated in the resistant strain (Fig. 4B). Analysis of eight putative secreted esterase-encoding transcripts [seven with identifiable signal-peptide sequences and one putative homolog of juvenile hormone esterase, which is also likely to be secreted (Fig. S1)] identified at least two, contigs CE3959 and CE21331, which were significantly up-regulated in the resistant strain (Fig. 4C).

**Figure 5:**
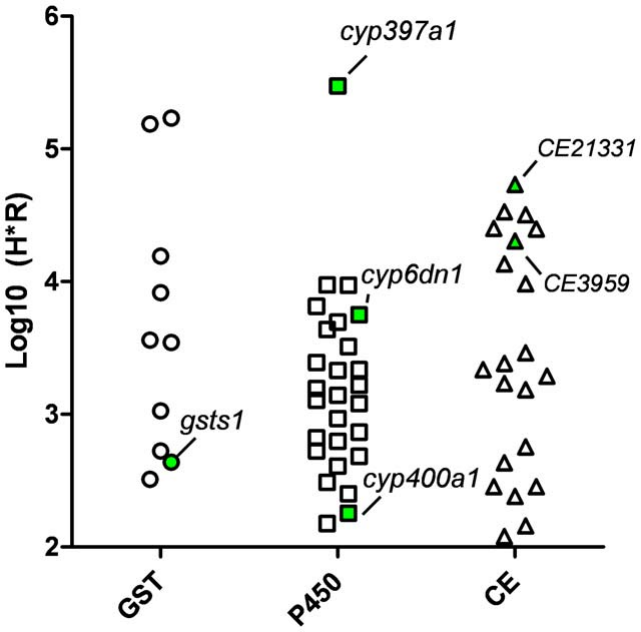
Relative abundance of metabolic genes. The product of the total number of reads from Harlan and Richmond 454 datasets used in the assembly of each P450, GST and CE contig was log_10_ transformed. GST, P450 and CE genes found to be significantly upregulated by qPCR in the resistant strain are highlighted in green.

Due to the relative nature of these assays, such qPCR data alone do not necessarily provide information as to the absolute abundance of the targeted transcripts. In order to independently determine which P450, GST and CE contigs were the most abundantly expressed in bed bugs, we plotted log_10_ values for the product of the number of 454 reads which mapped to each contig from Harlan and Richmond strain datasets (Fig. 5). Of the P450 genes, we found that transcripts derived from *cyp397a1* were by far the most abundant. As this gene also shows the greatest increase in transcriptional activity in Richmond strain bed bugs, these data imply that CYP397A1 may play the most prominent role in the cytochrome oxidase-mediated detoxification of pyrethroid insecticides. Likewise, *cyp6dn1* is among the most abundantly expressed of the remaining CYP450 gene contigs. Similarly, esterase-encoding contigs CE3959 (>6 fold), CE21331 (>4 fold) represented two of the six most abundant esterase transcripts in bed bugs, with CE21331 being the most abundant (Fig. 5). Consistent with the lack of increased GST activity observed in the model substrate assays, the upregulated contig GSTs1 was among the lowest expressed of the GSTs. Taken together, these data implicate several candidate gene products as potentially involved in pyrethroid metabolism.

## Discussion

We have identified bed bugs collected in Richmond, VA which exhibit both *kdr*-type and metabolic resistance to pyrethroid insecticides. The resistance ratios (∼5200-fold for deltamethrin and 111 for β-cyfluthrin) we quantified for this strain suggest that pyrethroid insecticides would be largely ineffective in controlling this multi-resistant population.

Pyrethroid insecticides target the voltage-gated sodium channel, with the sequence of the bed bug homolog reported in just 2008 [7]. We identified one of the two substitutions associated with *kdr* resistance as described by Yoon et al [7] in Richmond strain bed bugs. The identification of L925I, but not V419L substitutions in the voltage-gated sodium channel gene in this strain is consistent with a recent survey reported by Zhu et al [8], who found that 6 out of 7 strains (86%) collected from the state of Virginia carry this genotype. However, only 17 of the 110 populations they examined were screened for pyrethroid resistance, and the presence or strength of metabolic resistance in these strains was not addressed.

Several recent studies have indicated that different populations of bed bugs often have different mechanisms of resistance. While Yoon et al. [7] determined that the NY-BB strain did not exhibit enhanced metabolic enzyme activity, additional studies by Romero et al. [9] and Bai [10] do suggest that some bed bug populations may have enhanced metabolic activity as one of their resistance mechanisms. Additionally, a 2007 survey of the tropical bed bug (*Cimex hemipterus*), found that resistance to deltamethrin, permethrin, DDT, malathion, and propoxur was mainly the result of metabolic mechanisms, specifically CEs and GSTs [22]. Thus, we can expect that different bed bug populations within the U.S. and throughout the world may differ dramatically in their levels of resistance and resistance mechanisms, emphasizing the need for continuous surveillance.

As bed bugs were essentially absent from the U.S. for a number of decades, very little is known about the molecular biology or genetics of these ectoparasites. Using conventional Sanger sequencing of cDNA clones, Francischetti et al [23] recently reported an analysis of genes expressed and secreted into bed bug saliva. More recently, Bai et al [10] described a small 454 dataset from the Harlan bed bug strain (216,419 reads). While this group reported a number of P450 and GST sequences, the fragmented nature of their assembly (89% of contigs were singletons) makes their data concerning the number and phylogenic relationship of these sequences difficult to interpret. Our report adds another 2.5 million reads corresponding to both a long-time laboratory strain and a newly-collected field strain, including many high coverage, full-length descriptions of contigs encoding P450 and CE ORFs (both of which are typically 1.5–2 kb). These sequences should be of great assistance to future studies of insecticide resistance and, more generally, to studies of bed bug biology.

We observed a number of P450, GST and CE genes which were up-regulated in Richmond strain bed bugs compared to the pyrethroid-susceptible control strain. Four of the six up-regulated genes (*cyp397a1*, *cyp6dn1*, CE21331, *CE3959*) were at or among the most highly expressed of their respective classes, consistent with a prominent role in metabolic resistance to pyrethroid insecticides. We note that bed bugs, similar to another household pest, the German cockroach, have a history of being cross-resistant to many different substrates [24]. Therefore, these up-regulated genes may be indicators of past and potential future resistance to organophosphates and carbamates as well as pyrethroids. Of the bed bug genes identified in this study, CYP2, CYP3, CYP4 and CYP6 family members and the class I GSTs have all be shown to mediate detoxification functions in other insects, whereas the detoxification functions of orthologous CE genes are unclear [25], [26], [27]. Thus, future investigations directed at the *in vivo* metabolism of those pyrethroid and non-pyrethroid-based insecticides currently in use for bed bug control, as well as the relative *in vitro* substrate specificities of the overexpressed P450s, GSTs and CEs we describe here are warranted. We also observed several genes (*cyp400a1*, *gsts1*) which exhibited strong differential expression but were among the lowest expressed of their class. It is possible that these transcripts are abundantly expressed in a limited set of tissues, which would result in an overall low measure of expression when the entire bed bug is considered. Alternatively, these gene products may exhibit high selectivity for the target insecticide molecules. Thus we cannot necessarily rule out an important role for these gene products at this time.

Analysis and annotation of transcriptome sequences in the absence of a reference genome must ultimately face decision points regarding what is a separate gene, and what is polymorphism within the same gene. We were fairly conservative in treating near identical, but clearly variant sequences as a single gene. Thus, our analysis of the number of P450, GST and CE genes must be viewed as a minimum. This is especially true as our sequencing data came only from a single life stage (adult). Ultimately, only with a full bed bug genome sequence will it be possible to distinguish between highly similar genes such as P450s, which are well-known to undergo duplications and expansions in other insect species [28], [29].

In conclusion, our results indicate that highly-resistant bed bug populations can have multiple genetic mechanisms conferring resistance to pyrethroid (and possibly other) insecticides. In the case of the Richmond strain bed bugs, several forms of P450 and hydrolyzing esterases may be contributing to the bed bugs' overall ability to reduce their pesticide load. In addition, the alpha-subunit mutation reduces the potential for any non-metabolized pyrethroid to bind at the target site. It is reasonable to suggest that the genes responsible for these resistance mechanisms (*kdr* and enhanced metabolism) have been selected for in populations that have been subjected to long-term insecticide pressure.

## Supporting Information

Figure S1 **Carboxylesterase genes of the bed bug,** ***Cimex lectularius*** **.** Bed bug CEs (pink circle) were compared with published sequences from the pea aphid *Acyrthosiphon pisum* (gray diamond), the honey bee, *Apis mellifera* (green circle), and the vinegar fly, *Drosophila melanogaster* (purple triangle). Predicted protein sequences were aligned using ClustalW; tree construction was performed using the Neighbor-joining method in MEGA 4.0. All branches with less than 50% bootstrap support were collapsed, support greater than 80% is indicated (*). Clade designations followed the nomenclature of Oakeshott et al [30]. The presence (SP) or absence (-) of a predicted signal peptide is indicated, though this could not be determined (?) based on incomplete sequence information for all contigs. A putative juvenile hormone esterase homolog containing the GQSAG motif is indicated.
(TIF)

Table S1 **Primers used in this study.**
(DOC)
