# Supplementary figures and images for: Deep Sequencing of Pyrethroid-Resistant Bed Bugs Reveals Multiple Mechanisms of Resistance within a Single Population
Source: PLoS One. 2011 Oct 19;6(10):e26228. doi: 10.1371/journal.pone.0026228 (PMC3198472; doi:10.1371/journal.pone.0026228)

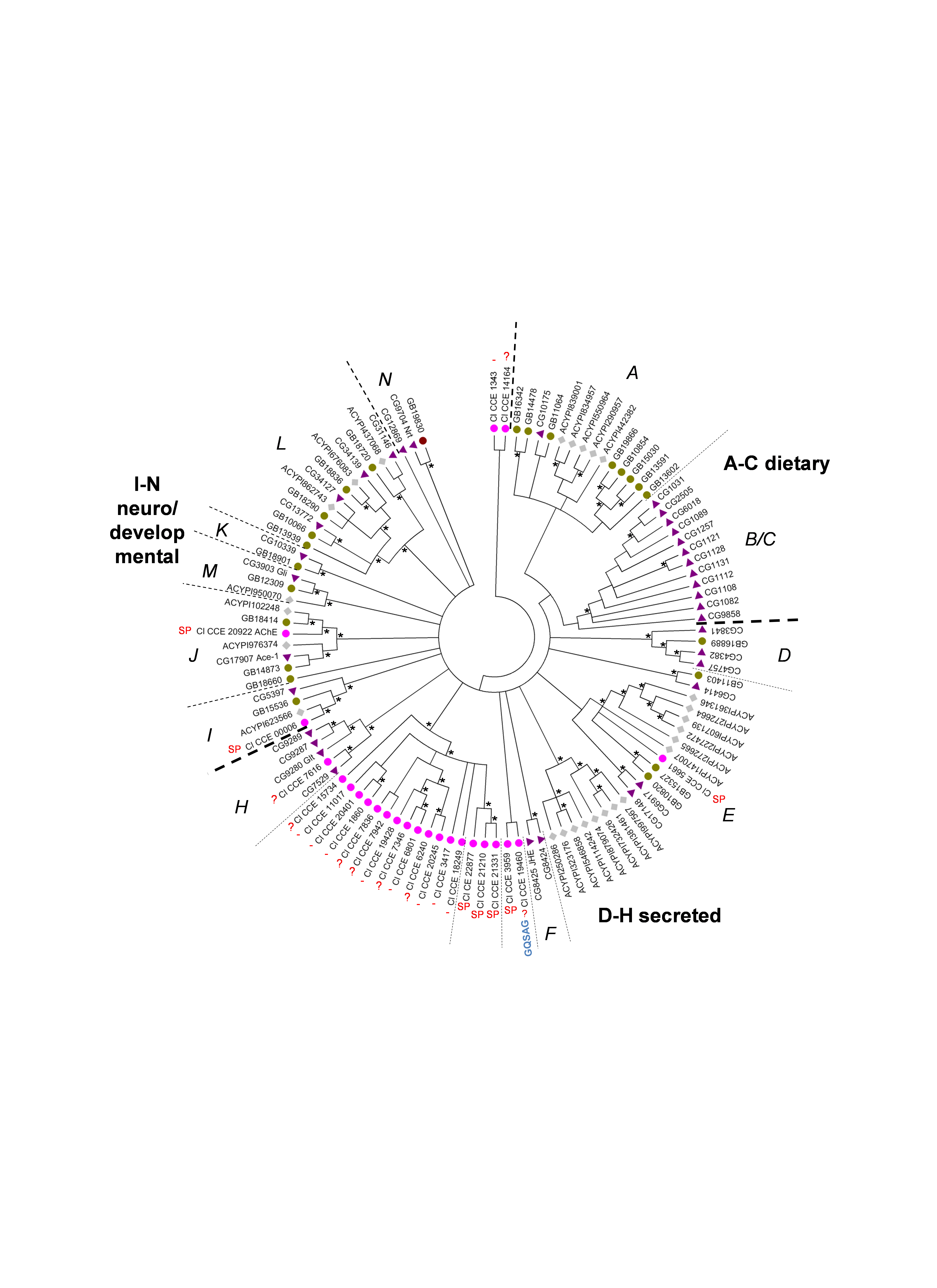

Supplement: Figure S1 — Carboxylesterase genes of the bed bug, Cimex lectularius . Bed bug CEs (pink circle) were compared with published sequences from the pea aphid Acyrthosiphon pisum (gray diamond), the honey bee, Apis mellifera (green circle), and the vinegar fly, Drosophila melanogaster (purple triangle). Predicted protein sequences were aligned using ClustalW; tree construction was performed using the Neighbor-joining method in MEGA 4.0. All branches with less than 50% bootstrap support were collapsed, support greater than 80% is indicated (*). Clade designations followed the nomenclature of Oakeshott et al [30]. The presence (SP) or absence (-) of a predicted signal peptide is indicated, though this could not be determined (?) based on incomplete sequence information for all contigs. A putative juvenile hormone esterase homolog containing the GQSAG motif is indicated. (TIF) [file pone.0026228.s001.tif]
