# Supplementary material for: Deep Sequencing of Pyrethroid-Resistant Bed Bugs Reveals Multiple Mechanisms of Resistance within a Single Population
Source: PLoS One. 2011 Oct 19;6(10):e26228. doi: 10.1371/journal.pone.0026228 (PMC3198472; doi:10.1371/journal.pone.0026228)
Supplement: Table S1 — Primers used in this study. (DOC) [file pone.0026228.s002.doc]

Table S1. Primers used for quantitative real-time PCR experiments.

| Control genes: | | F (5' --> 3') |  | R (5' --> 3') |
| --- | --- | --- | --- | --- |
| *α-tubulin* | GTGTGCGATGAAAGGGAATGA | |  | TCGGGAGCAGAGGGGAGTA |
| *myosin* | TTCAAGTTTGGCGGTAAGAGA | |  | GAAAAGGCAAAGAGCGAGACT |
|  |  | |  |  |
| *GSTs:* |  | |  |  |
| *s1* | AGGAGAGCCAGTTAGATTTATGTT | |  | AAGCGATTCCCACCGATTTT |
| *s2* | TGCGCTAAGTACACAAACATCAAG | |  | CGAACATTTACGCATATCAACTAC |
| *s3* | TGGCCGCAGTACAAGGAGTCTAT | |  | TTCAACGGCCATGTCAATCTGTA |
| *s4* | AGCGCACATTAACCGACCTG | |  | GAAGAATGGCAGAAGTTAAAGCAA |
| *s5* | GCATTGGCCAACAGCTTTCTTTA | |  | CTGTGGCCATGGCAGTCTTA |
| *o1* | GATAATCTGCGACTTCCTTGACGA | |  | CATGCCCGGTTTGCTTCCTGA |
| *z1* | TCGGGCTCGGGGTAGGAAACT | |  | CGGGGCGTCGGACAACATTA |
| *d1* | GCGGAGCCTCTTATGATGAA | |  | GCCCTCTTGATTAGCCTCCTT |
| *t1* | ATAGACGATGATGGCTTTGTTGTA | |  | CTTGGAATTTGGAGCGACTTTAG |
| unk1 (z2) | ATTTTTCGGGCCGTTCTTCTATTT | |  | CATGGAGGGCGAGTGTGACC |
|  |  | |  |  |
| *P450s:* |  | |  |  |
| *cyp6dm1* | ATGAATTTCGTACCATGGCAGATA | |  | AACGAATTTTAAACCGAGAACTCC |
| *cyp6dm2* | CCCCCTTATGCTACCCGTTTGA | |  | TTCGTCCTTTTTATGTCCGTCTGC |
| *cyp6dn1* | AGCCCACAGCTCGGAAACAG | |  | CTTATGCCTTGGTGGGAGAGT |
| *cyp6dn2* | TAAAACTAGGGGACAAAGCAAAAT | |  | TAAAACGGATAAAACACGCACAT |
| *cyp6dn3* | TGGAGGCGATAACATGGACTTTG | |  | TTCGGGTATTCTTTGTATTGGTTG |
| *cyp6dn4* | CCCAAACTACAAATTCATCGTCAT | |  | TCCCAAATTTACTATATCCCTGTC |
| *cyp396a1* | TCTGTTCGGAGGACTGCTGTATTA | |  | CGGGGTCGGTGAAGTTGTAGTATC |
| *cyp397a1* | CTCGGGCTCACCACTCTCAACA | |  | ACCGTCATGGCTCCCGTCAG |
| *cyp398a1* | AATCGCCCACAGGAAAAACAACAA | |  | CCGGGTGGGAAGCGAGTAGG |
| *cyp399a1* | CAATTTGGCCCTTTTCTGTGC | |  | TAATGGGCCTTTTCCTTGTTGTAG |
| *cyp400a1* | CCTGCGCGTTCGGAGTCAATA | |  | CATCGGCTAAATAGAGGAAAAAGT |
| *cyp4g52* | TCCACCTCCAGGCACTATTATTCT | |  | TAGGCCTAGTGAGCTTTCTGGTGT |
| *cyp4g53* | ACACGACGGCAGCAGGCAGTAGT | |  | GGCGGGCACAGGAGGAAAAA |
| *cyp4g54* | GGCAATTCCGACCGCGATGTGAC | |  | GCAGCCAGCAGGGACGACCAA |
| *cyp4cm1* | AGGAGAATGTCAGAAAGCGTCACC | |  | GCATTAATTTTTCCCAACTTTCTT |
| *cyp4cn1* | AACGGTGCCATTTATCGGGAGGAA | |  | CTGCACTGAATGGGATGAAACTGA |
| *cyp4cp1* | GGACTGCACTCTGGACATTATTTG | |  | CCTCGACTTCTTTGGGTGATTTGA |
| *cyp4cq1* | GGTGGCATTTTGGTTGGGTCCTGT | |  | TAAAATGAAATGTTGGCGTGAGCA |
|  |  | |  |  |
| *CEs:* |  | |  |  |
| *CE_5661* | CTCGGGCCACTGGGTTTCCTGA | |  | GCTGGCTGCCCCTGCTGACAT |
| *CE_3959*  *CE_19460* | ACGTCTGGAGAAGGGCAACTGAAA  ATGGGCTGCGACGACTATGGATG | |  | GACGGCCGGGTAGATGAAAACAAC  AAGAGTTGACACCGGTGAGGATGG |
| *CE_21331* | TCTCACGGGGACGAACTGCCTTAT | |  | CCTGGTCTTCTGGGTATTTCTTCA |
| *CE_21210* | GGTTCGAGCCAGGGCAGCATTTA | |  | AAGCCGTGACCCCCGAGTGTTATC |
| *CE_22877*  *CE_00006*  *CE_20922* | CAACGGTCGCATGGCAGGTAAGAT  GTCCAGCTTCCTCGGCATCAGA  CGTGGGGAGCAACAAGGAAG | |  | TGGGCAGAGACGTAATGGTTTTGA  GGCGTAAAGAATCACCACCAGTTG  TCGCCGACCATTTTATCAAGAG |
